# Association between prehospital vitamin D status and incident acute respiratory failure in critically ill patients: a retrospective cohort study

**DOI:** 10.1136/bmjresp-2014-000074

**Published:** 2015-06-13

**Authors:** David R Thickett, Takuhiro Moromizato, Augusto A Litonjua, Karin Amrein, Sadeq A Quraishi, Kathleen A Lee-Sarwar, Kris M Mogensen, Steven W Purtle, Fiona K Gibbons, Carlos A Camargo, Edward Giovannucci, Kenneth B Christopher

**Affiliations:** 1 School of Clinical and Experimental Medicine, College of Medical and Dental Sciences, University of Birmingham, Birmingham, UK; 2 Department of Medicine, Hokubu Prefectural Hospital, Nago City, Japan; 3 Channing Division of Network Medicine and Pulmonary and Critical Care Division, Department of Medicine, Brigham and Women's Hospital, Boston, Massachusetts, USA; 4 Division of Endocrinology and Metabolism, Department of Internal Medicine, Medical University of Graz, Graz, Austria; 5 Department of Anesthesia, Critical Care and Pain Medicine, Massachusetts General Hospital, Boston, Massachusetts, USA; 6 Department of Medicine, Brigham and Women's Hospital, Boston, Massachusetts, USA; 7 Department of Nutrition, Brigham and Women's Hospital, Boston, Massachusetts, USA; 8 Division of Pulmonary Sciences and Critical Care Medicine, University of Colorado, Denver, Colorado, USA; 9 Division of Pulmonary and Critical Care Medicine, Department of Medicine, Massachusetts General Hospital, Boston, Massachusetts, USA; 10 Department of Emergency Medicine, Massachusetts General Hospital, Boston, Massachusetts, USA; 11 Departments of Nutrition and Epidemiology, Harvard School of Public Health, Boston, Massachusetts, USA; 12 The Nathan E. Hellman Memorial Laboratory, Renal Division, Brigham and Women's Hospital, Boston, Massachusetts, USA

**Keywords:** ARDS, Clinical Epidemiology

## Abstract

**Objective:**

We hypothesise that low 25-hydroxyvitamin D (25(OH)D) levels before hospitalisation are associated with increased risk of acute respiratory failure.

**Design:**

Retrospective cohort study.

**Setting:**

Medical and Surgical Intensive care units of two Boston teaching hospitals.

**Patients:**

1985 critically ill adults admitted between 1998 and 2011.

**Interventions:**

None.

**Measurements and main results:**

The exposure of interest was prehospital serum 25(OH)D categorised as ≤10 ng/mL, 11–19.9 ng/mL, 20–29.9 ng/mL and ≥30 ng/mL. The primary outcome was acute respiratory failure excluding congestive heart failure determined by International Classification of Diseases Ninth Edition (ICD-9) coding and validated against the Berlin Definition of acute respiratory sistress syndrome. Association between 25(OH)D and acute respiratory failure was assessed using logistic regression, while adjusting for age, race, sex, Deyo-Charlson Index and patient type (medical vs surgical).

In the cohort, the mean age was 63 years, 45% were male and 80% were white; 25(OH)D was ≤10 ng/mL in 8% of patients, 11–19.9 ng/mL in 24%, 20–29.9 ng/mL in 24% and ≥30 ng/mL in 44% of patients. Eighteen per cent (n=351) were diagnosed with acute respiratory failure. Compared to patients with 25(OH)D ≥30 ng/mL, patients with lower 25(OH)D levels had significantly higher adjusted odds of acute respiratory failure (≤10 ng/mL, OR=1.84 (95% CI 1.22 to 2.77); 11–19.9 ng/mL, OR=1.60 (95% CI 1.19 to 2.15); 20–29.9 ng/mL, OR=1.37 (95% CI 1.01 to 1.86)).

**Conclusions:**

Prehospital 25(OH)D was associated with the risk of acute respiratory failure in our critically ill patient cohort.

**Key messages**

- Vitamin D deficiency prior to hospitalization is associated with development of acute respiratory failure.
- Vitamin D deficient patients who develop acute respiratory failure have heightened mortality.

## Introduction

In the USA, every year, 190 600 patients develop acute respiratory distress syndrome (ARDS), with 74 500 associated deaths and 2.2 million days in the intensive care unit (ICU).^1^ Incidences of ARDS among patients with acute illness and mechanically ventilated patients are 7% and 11–23%, respectively.^2^ Although critical care outcomes in general and those associated with acute respiratory failure have improved over time, long-term outcomes of ICU survivors has gained prominence.^3^ ^4^ Among the more than 100 000 patients who survive ARDS each year, many develop cognitive abnormalities, depression, post-traumatic stress disorders and have poor health-related quality of life.^1^ ^5^

Recent evidence suggests that vitamin D is a key regulator of the innate and adaptive immune system.^5^ Serum 25-hydroxyvitamin D (25(OH)D) is the major circulating metabolite of vitamin D, the standard measure of vitamin D status and is used to assess therapeutic response to supplementation. Low serum 25(OH)D levels are associated with increased risk of viral and bacterial infections as well as sepsis possibly due to effects on innate and adaptive immunity.^6^ ^7^ Additionally, vitamin D modulates inflammation, fibrosis and airway destruction in the lung which are major steps in ARDS pathogenesis.^8^ ^9^ Further, decreased muscle strength and mass is associated with vitamin D deficiency.^10^ Recent data from a randomised controlled trial on high-dose vitamin D in critically ill patients (VITdAL-ICU trial) shows as a secondary outcome the improved survival in patients with severe vitamin D deficiency (25(OH)D≤12 ng/mL) who receive vitamin D.^11^ Data are lacking with regards to the association of prehospitalisation vitamin D inadequacy and incident ARDS during critical illness.

Given that vitamin D inadequacy, defined by measuring circulating 25(OH) D_3_ levels, is increasingly prevalent in the general population of the USA and associated with critical care outcomes, we performed a two-centre observational study of adult patients among whom 25(OH)D concentrations had been measured for routine clinical reasons within 1 year before hospitalisation.^7^ ^12–23^ The objective of this study was to test the hypothesis that vitamin D status before hospital admission is inversely associated with the risk of developing acute respiratory failure in a critically-ill patient cohort.

## Materials and methods

Data on all patients admitted to two teaching hospitals in Boston between 4 August 1998 and 12 January 2011 were obtained through a computerised registry. During the study period, there were 79 927 patient ICU admissions to the hospitals under study. Approval for the study was granted by the Partners Human Research Committee (Institutional Review Board). Requirement for consent was waived as the data were analysed anonymously. The cohort included patients ≥18 years of age who received critical care and had serum 25(OH)D measured between 7 and 365 days before hospitalisation. We excluded patients with congestive heart failure (International Classification of Diseases Ninth Edition (ICD-9) 428.0–428.9) diagnosed following hospital admission.^24^ The exposure of interest was serum 25(OH)D level obtained 7 to 365 days prior to the date of hospital admission and categorised as 25(OH)D <10 ng/mL; 10–19.9 ng/mL; 20–29.9 ng/mL and ≥30 ng/mL.^14^ We define prehospital serum 25(OH)D concentration <20 ng/mL as vitamin D inadequacy, as suggested by a recent Institute of Medicine report.^25^ In cases where a patient had serum 25(OH)D measured more than once in the year prior to hospitalisation, the serum 25(OH)D measured closest to the date of hospital admission was utilised.

Race was either self-determined or designated by a patient representative/healthcare proxy. Patient admission ‘type’ was defined as ‘medical’ or ‘surgical’ and incorporates the Diagnostic-Related Grouping (DRG) methodology.^26^ We utilised the Deyo-Charlson Index to assess the burden of chronic illness which is well studied and validated.^27^ Sepsis was defined by the presence of any of the following ICD-9-clinical modification (CM) codes 038.0–038.9, 020.0, 790.7, 117.9, 112.5 or 112.81^28^, 3 days prior to critical care initiation to 7 days after critical care initiation, a definition validated in our administrative data.^7^ To determine neighbourhood socioeconomic disadvantage we used geocoded residential address data^29^ from electronic health records; we then linked the zip+4 data to the Area Deprivation Index developed by Singh *et al*^30^ and linked to the 2000 US census by Kind *et al.*^31^

During the study period between 1998 and 2011, the chemiluminescence assay, the radioimmunoassay or liquid chromatography-mass spectroscopy (LC-MS) were employed at different times as a 25(OH)D assay method. Dates, times and type of 25(OH)D assay were recorded. The 25(OH)D assays were tested for imprecision by the clinical laboratories at the two hospitals. Imprecision testing with human serum specimens showed within-run coefficients of variation (CVs) of ≤4.5% for the chemiluminescence assay, ≤10.8% for the radioimmunoassay, and ≤8.6% for LC-MS.^6^

The primary end point was acute respiratory failure defined by Cooke *et al*^24^ and identified by the presence of ICD-9, codes for respiratory failure or pulmonary oedema (518.4, 518.5, 518.81 and 518.82) and mechanical ventilation (96.7×), excluding congestive heart failure (428.0–428.9) following hospital admission.^24^ Inclusion of mechanical ventilation codes and the exclusion of heart failure codes increases the specificity of the ICD-9 code combination for ARDS.^32^ In subanalyses, the acute respiratory failure end point was validated against the Berlin Definition of ARDS.^33^ The secondary end point was 90-day all-cause mortality obtained from the Social Security Administration Death Master File. One hundred per cent of the cohort had at least 90-day follow-up. The censoring date was 5 January 2012.

### Power calculations and statistical analysis

In the cohort under study, the incidence of acute respiratory failure was 18%. By assuming acute respiratory failure incidence in patients with prehospital serum 25(OH)D >30 ng/mL to be 18% and an OR of 1.5 for incident acute respiratory failure in patients with prehospital serum 25(OH)D ≤30 ng/mL, we needed 359 patients with 25(OH)D >30 ng/mL and 359 patients with 25(OH)D ≤30 ng/mL to achieve 80% power and 5% significance.

Unadjusted associations between vitamin D groups and outcomes were estimated by bivariable logistic regression analysis. Adjusted ORs were estimated by multivariable logistic regression models with inclusion of covariate terms chosen based on the biological plausibility of possible confounding of the vitamin D—acute respiratory failure association. For the primary model, specification of each continuous covariate was adjudicated by the empiric association with the primary outcome using Akaike's Information Criterion; the overall model fit was assessed using the Hosmer Lemeshow test. Models for secondary analyses (90-day mortality) were specified identically to the primary model in order to achieve greatest analogy. Sensitivity analysis were performed for patients with 25(OH)D measured at various time points prior to hospital admission. Locally weighted scatter plot smoothing (LOWESS) was used to graphically represent the relationship between prehospital 25(OH)D concentration and rate of acute respiratory failure. A multivariable Cox’s proportional hazards model was used to illustrate the survival among patients with acute respiratory failure as vitamin D intake increases. All p values presented are two-tailed; values below 0.05 were considered to be significant. All analyses are performed using STATA 12.0MP (College Station, Texas, USA).

To validate the accuracy of ICD-9-CM and current procedural terminology (CPT)-defined acute respiratory failure assignment in our study, 206 of the 1985 cohort patients were chosen at random. Subject charts were retrospectively reviewed by two clinician investigators blinded to the subject 25(OH)D level and ICD-9-CM/CPT acute respiratory failure code assignment to determine if patients had clinical criteria for ARDS in the first 14 days of an ICU admission according to the Berlin definition: (1) acute respiratory failure not fully explained by cardiac failure or fluid overload, per the intensivist of record; (2) bilateral opacities consistent with pulmonary oedema on the chest radiograph or the CT scan; and (3) onset within 1 week after a known clinical insult or new or worsening respiratory symptoms.^33^ The validation criteria were met if the criteria for mild, moderate or severe ARDS, as defined by The Berlin Definition of ARDS, were achieved in the first 14 days of an ICU admission: mild if PaO_2_/FiO_2_=201–300 mm Hg, moderate if PaO_2_/FiO_2_=101–200 mm Hg and severe if PaO_2_/FiO_2_ ≤100 mm Hg with PEEP level ≥5 cm H_2_O was present in all ARDS cases. Diagnosis of ARDS was established by consensus of the two clinician investigators and resolved by a third in case of discrepancies.

## Results

Table 1 shows demographic characteristics of the parent ICU cohort and the study cohort. Differences between the parent and study cohorts included gender, patient type and comorbidity. In the study cohort (N=1985), most patients were women, white and had medical-related DRG with a mean age of 63 years. The mean (SD) 25(OH)D in the study cohort was 29.0 (15.5) ng/mL which did not statistically differ by season of 25(OH)D draw (p=0.12, χ^2^). The majority of 25(OH)D measurements in the study cohort occurred within 6 months of hospital admission (20% within 1 month, 48% within 3 months and 72% within 6 months). Most study cohort patients had 25(OH)D levels ≥20 ng/mL (8% with 25(OH)D <10 ng/mL; 24% with 10–19.9 ng/mL; 24% with 20–29.9 ng/mL and 44% with ≥30 ng/mL). The 90-day mortality of the study cohort was 15.7%.

**Table 1:** Characteristics of total ICU cohort (N=77 927) and study cohort (N=1985)

	Total ICU cohort	Study cohort
N	79 927	1985
*Age-mean(SD)*	61.8 (18.3)	63.2 (16.2)
Sex number (%)		
Female	33 556 (42)	1083 (55)
Male	46 371 (58)	902 (45)
Race number (%)		
White	63 712 (80)	1602 (81)
Non-white	16 215 (20)	383 (19)
Patient type number (%)		
Medical	39 355 (49)	1372 (69)
Surgical	40 572 (51)	613 (31)
Deyo-charlson index number (%)		
0–3	56 312 (70)	1227 (62)
4–6	19 060 (24)	553 (28)
>6	4555 (6)	205 (10)
Body mass index-mean(SD)*	27.3 (6.9)	27.3 (7.7)
Area deprivation index-mean(SD)†	86.0 (32.8)	81.8 (30.4)
Sepsis number (%)	10 215 (13)	231 (12)
Acute respiratory failure number (%)	12 308 (15)	351 (18)
90-day mortality number (%)	13 860 (17)	311 (16)

Greater Area Deprivation Index means a greater disadvantage.

*5527 of the total ICU cohort and 435 study cohort patients had body mass index determined.

†Area Deprivation Index is an ecological measure of socioeconomic disadvantage.

ICU, intensive care unit.

In the study cohort, the most common Major Diagnostic Codes in the cohort by system were circulatory 31.7%, digestive 14%, musculoskeletal 8.9%, kidney 8.4%, endocrine 7.7%, respiratory 4.8%, injuries 4.4% and infectious 4.2%. The mean (SD) time from hospital admission to acute respiratory failure diagnosis was 1.46 (4.43) days and the mean (SD) time from hospital admission to ICU admission was 1.90 (6.19) days. Validation of ICD-9-CM/CPT-defined acute respiratory failure assignment, as per the Berlin Definition of ARDS, showed that ICD-9-CM/CPT-defined acute respiratory failure assignment had a sensitivity of 59.4% (95% CI 46.4 to 71.5%), a specificity of 95.1% (95% CI 90.1 to 97.9%), a positive predictive value of 84.4% (95% CI 69.9 to 93.0) and a negative predictive value of 83.9% (95% CI 77.0 to 89.0).

Patient characteristics of the study cohort were stratified according to preadmission vitamin D groups (table 2). Factors that significantly differed between stratified groups included age, gender, calcium and haematocrit. Factors that did not significantly differ between stratified groups included Deyo-Charlson index, patient type (surgical vs medical) and season of 25(OH)D measurement. Table 3 indicates that age, gender and 25(OH)D are significant predictors of acute respiratory failure in our model.

**Table 2:** Patient characteristics by prehospital vitamin D status

	Prehospital 25(OH)D, ng/mL					
	<10.0	10–19.9	20–29.9	≥30	Total	
N	164	477	473	871	1985	p Value
*Age-mean (SD)*	57.3 (16.9)	61.3 (16.6)	62.4 (15.9)	65.9 (15.4)	63.2 (16.2)	0.001*
Sex number (%)						0.01
Female	83 (51)	243 (51)	246 (52)	511 (59)	1083 (55)	
Male	81 (49)	234 (49)	227 (48)	360 (41)	902 (45)	
Race number (%)						<0.0001
White	125 (76)	366 (77)	371 (78)	740 (85)	1602 (81)	
Non-white	39 (24)	111 (23)	102 (22)	39 (15)	383 (19)	
African–American	17 (10)	43 (9)	34 (7)	34 (4)	128 (6)	
Patient type number (%)						0.27
Medical	115 (70)	343 (72)	331 (70)	583 (67)	1372 (69)	
Surgical	49 (30)	134 (28)	142 (30)	288 (33)	613 (31)	
Deyo-Charlson index number (%)						0.087
0–3	88 (54)	285 (60)	294 (62)	560 (64)	1227 (62)	
4–6	59 (36)	135 (28)	137 (29)	222 (25)	553 (28)	
>6	17 (10)	57 (12)	42 (9)	89 (10)	205 (10)	
Diabetes mellitus number (%)	49 (30)	193 (40)	159 (34)	274 (31)	675 (34)	0.005
Body mass index-mean (SD)†	27.9 (8.2)	28.1 (9.9)	28.0 (6.5)	25.9 (5.9)	27.3 (7.7)	0.064*
Season of 25(OH)D draw number (%)						0.18
Spring	43 (26)	132 (28)	141 (30)	242 (28)	558 (28)	
Summer	35 (21)	114 (24)	120 (25)	229 (26)	498 (25)	
Winter	38 (23)	119 (25)	103 (22)	162 (19)	422 (21)	
Fall	48 (29)	112 (23)	109 (23)	238 (27)	507 (26)	
Calcium ≥10.5 mg/dL number (%)	26 (16)	66 (14)	60 (13)	74 (9)	226 (11)	0.003
Haematocrit <30% number (%)	31 (19)	98 (21)	88 (19)	121 (14)	338 (17)	0.009
>90 days between 25(OH)D and Admission number (%)	56 (34)	192 (40)	264 (56)	516 (59)	1028 (52)	<0.001
Area deprivation index-mean (SD)	87.1 (28.1)	84.6 (27.1)	84.1 (28.3)	77.9 (33.2)	81.8 (30.4)	<0.001*
Sepsis number (%)	28 (17)	73 (15)	53 (11)	77 (9)	231 (12)	0.001
Sputum culture positivity number (%)	30 (21)	92 (22)	71 (17)	119 (15)	312 (17)	0.01
Acute respiratory failure number (%)	41 (25)	102 (21)	89 (19)	119 (14)	351 (18)	<0.001
90-day mortality number (%)	34 (21)	111 (23)	76 (16)	90 (10)	311 (16)	<0.001

p Values determined by χ^2^ unless designated by (*) then p value determined by ANOVA.

Columns may not add up to 100% due to rounding. Greater Area Deprivation Index means a greater disadvantage.

†435 cohort patients had body mass index determined.

**Table 3:** Multivariable-adjusted associations between covariates and acute respiratory failure

	Acute respiratory failure Absent* 1634	Acute respiratory failure Present* 352	p Value†	OR‡	95% CI	p Value
*Age (per 1 year)*	64 (16)	59 (17)	<0.001	0.99	0.98 to 0.99	<0.001
Sex			0.002			
Male	716	186		1.37	1.09 to 1.74	0.008
Female	918	165		1	Referent	
Race			0.22			
Non-white	307 (19)	76 (22)		1.10	0.82 to 1.47	0.53
White	1327 (81)	275 (78)		1	Referent	
Patient type			0.14			
Surgical	493 (70)	120 (66)		1.22	0.95 to 1.57	0.11
Medical	1141 (30)	231 (34)		1	Referent	
Deyo-Charlson index			0.012			
0–3	1034 (63)	193 (55)		1	Referent	
4–6	435 (27)	118 (34)		1.45	1.13 to 1.88	0.004
≥6	165 (10)	40 (11)		1.30	0.89 to 1.90	0.18
25(OH)D§			<0.001			
<10 ng/mL	123 (8)	41 (12)		1.84	1.22 to 2.77	0.004
10–19.9 ng/mL	375 (23)	102 (29)		1.60	1.19 to 2.15	0.002
20–29.9 ng/mL	384 (24)	89 (25)		1.37	1.01 to 1.86	0.04
≥30 ng/mL	752 (46)	119 (34)		1.00	Referent	

Estimates for each variable are adjusted for all other variables in the table.

*Number (%) shown except for age which is shown as Mean (SD).

†p Values determined by χ^2^ except age determined by Kruskal-Wallis.

‡OR for acute respiratory failure.

§25(OH)D estimates adjusted for age, race, sex, Deyo-Charlson Index and patient type (medical vs surgical).

### Primary outcome

**Figure 1:**
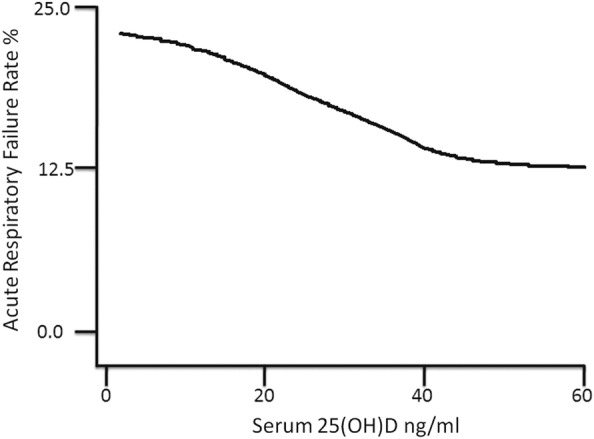
Vitamin D status versus acute respiratory failure Rate. 25(OH)D=25-hydroxyvitamin D. Locally weighted scatter plot smoothing (LOWESS) utilised to represent the near inverse linear association between prehospital 25(OH)D concentration and acute respiratory failure rate. With bandwidth parameter=0.99, 1919 cohort patients were utilised to construct the curve.

Preadmission 25(OH)D was strongly associated with acute respiratory failure during hospitalisation (table 4). The odds of acute respiratory failure was 2.1-fold, 1.7-fold and 1.5-fold higher in patients with 25(OH)D values in the <10 ng/mL, 10–19.9 ng/mL, 20–29.9 ng/mL groups, respectively, compared to those with 25(OH)D ≥30 ng/mL. 25(OH)D in the cohort remains a significant predictor of odds of acute respiratory failure following adjustment for age, race, sex, Deyo-Charlson Index and patient type (medical vs surgical). The adjusted odds of acute respiratory failure was 1.8-fold, 1.6-fold and 1.4-fold higher in patients with 25(OH)D values in the <10 ng/mL, 10–19.9 ng/mL, 20–29.9 ng/mL groups, respectively, compared to those with 25(OH)D ≥30 ng/mL (table 4). LOWESS plot (figure 1) showed a near inverse linear association between vitamin D status and an acute respiratory failure rate for 25(OH)D concentrations of approximately 40 ng/mL; beyond 40 ng/mL, the association appears flat.

**Table 4:** Unadjusted and adjusted associations between prehospital 25(OH)D level and acute respiratory failure

	OR	95% CI	p Value
*Unadjusted*			
25(OH)D			
<10 ng/mL	2.11	1.41 to 3.15	<0.0001
10–19.9 ng/mL	1.72	1.28 to 2.30	<0.0001
20–29.9 ng/mL	1.46	1.08 to 1.98	0.01
≥30 ng/mL	1.00	Referent	
*Adjusted*			
25(OH)D			
<10 ng/mL	1.84	1.22 to 2.77	0.004
10–19.9 ng/mL	1.60	1.19 to 2.15	0.002
20–29.9 ng/mL	1.37	1.01 to 1.86	0.04
≥30 ng/mL	1.00	Referent	

CI, OR for acute respiratory failure.

Referent in each case is 25(OH)D ≥30 ng/mL.

Estimates adjusted for age, race, sex, Deyo-Charlson Index and patient type (medical vs surgical).

Individually running the adjusted model with and without terms for assay, year, season or sepsis did not materially affect the acute respiratory failure estimates reported above (see online supplementary table S1); thus, the vitamin D-acute respiratory failure relationship was not materially confounded by these covariates. Further, utilising 25(OH)D as a continuous variable, we find that for every increase in preadmission 25(OH)D of 5 ng/mL, the odds of acute respiratory failure during hospitalisation decreases by 8% (OR 0.92, 95% CI 0.88 to 0.96; p<0.001) when adjusted for age, gender, race, type and Deyo-Charlson index.

We also performed a sensitivity analysis of the timing of prehospital 25(OH)D draw date. Sensitivity analysis of the effects of excluding patients with preadmission 25(OH)D levels obtained ≥30, ≥60, ≥90 or ≥180 days prior to admission did not materially change the association between vitamin D and ICD-9-CM defined acute respiratory failure (see online supplementary table S2). A modest attenuation of the effect size was observed as the time difference increased, but the vitamin D acute respiratory failure associations retained statistical significance.

In cohort patients with acute respiratory failure (n=381), vitamin D status was associated with risk of mortality. Though limited by sample size, patients with prehospital 25(OH)D values of <10 ng/mL, 10–19.9 ng/mL and 20–29.9 ng/mL had odds of 90-day mortality of 2.02 (95% CI 0.96 to 4.27; p=0.064), 2.57 (95% CI 1.49 to 4.45; p<0.001) and 1.57 (95% CI 0.89 to 2.80; p=0.12), respectively, compared to those with 25(OH)D ≥30 ng/mL following adjustments for age, race, sex, Deyo-Charlson Index and patient type (medical vs surgical; table 5). Cox proportional-hazards regression analysis demonstrated that in patients with acute respiratory failure, the risk of death declines with each 5 ng/mL increase in prehospital 25(OH)D adjusted for age, race, sex, Deyo-Charlson Index and patient type (medical vs surgical; HR 0.92, 95% CI 0.87 to 0.97; p=0.004).

**Table 5:** Unadjusted and adjusted associations between prehospital 25(OH)D level and 90-day mortality in patients with acute respiratory failure

	OR	95% CI	p Value
*Unadjusted*			
25(OH)D			
<20 ng/mL	2.19	1.31 to 3.63	0.003
20–29.9 ng/mL	1.37	0.77 to 2.44	0.285
≥30 ng/mL	1.00	Referent	
*Adjusted*			
25(OH)D			
<20 ng/mL	2.48	1.45 to 4.25	0.001
20–29.9 ng/mL	1.48	0.82 to 2.70	0.195
≥30 ng/mL	1.00	Referent	

CI, OR for 90-day mortality. N=381, 90-day mortality 39.9%. Referent in each case is 25(OH)D ≥30 ng/mL.

<20 ng/mL cut point chosen for power issues. Estimates adjusted for age, race, sex, Deyo-Charlson Index and patient type (medical vs surgical). 100% of the cohort had vital status follow-up at 90 days.

### Effect modification

Analyses based on fully-adjusted models were performed to evaluate the 25(OH)D-acute respiratory failure association, and p for interaction was determined to explore for any evidence of effect modification. We individually tested for effect modification by hospital, time between 25(OH)D draw and hospital admission and season of 25(OH)D draw by adding an interaction term to the multivariate models. Effect modification analysis showed that the association between 25(OH)D and acute respiratory failure was not modified by the hospital that provided care (p interaction=0.19), or the time between 25(OH)D draw and hospital admission (p interaction=0.30). When we investigated effect modification by season of 25(OH)D draw, we identified a nearly statistically significant interaction term for 25(OH)D and acute respiratory failure (p interaction=0.07). When the cohort is analysed by restricting this to the season of 25(OH)D draw, the directionality of the association between 25(OH)D and acute respiratory failure remains, but the effect size is strongest with 25(OH)D drawn in the spring season and weakest in the fall.

## Discussion

The present, two-centre study aimed to determine whether suboptimal vitamin D status prior to hospital admission was associated with acute respiratory failure in the critically ill. In unadjusted and adjusted analyses, we found increased odds of acute respiratory failure in patients with preadmission 25(OH)D <20 ng/mL. In addition, our mortality analysis suggests that patients with 25(OH)D levels <20 ng/mL before hospital admission who develop acute respiratory failure have a higher risk for mortality compared to patients with prehospital 25(OH)D levels ≥30 ng/mL.

ARDS is due to diffuse alveolar damage mediated by inflammatory cytokines such as tumour-necrosis factor, interleukin (IL)-1, IL-6 and IL-8 and subsequent neutrophil recruitment and release of oxygen free radicals that damage capillary endothelium and alveolar epithelium.^34^ ^35^ Immunomodulatory and proinflammatory associations of vitamin D deficiency are well known. Vitamin D induces expression of the gene for cathelicidin, which promotes intracellular killing of bacteria.^36^ In the lung, cathelicidin likely plays a role in mucosal defence as it is produced by neutrophils, macrophages and airway epithelium, and upregulated in response to infection and inflammation.^37^ These biological observations indicate the potential importance of vitamin D status to innate immunity and ARDS.

Prior studies have reported robust associations of low vitamin D and sepsis, bloodstream infections and mortality in critically ill adults.^13^ The VITdAL-ICU trial secondary outcome data shows high-dose vitamin D supplementation reduces mortality in patients with severe vitamin D deficiency (25(OH)D≤12 ng/mL), but was not designed to study respiratory outcomes.^11^ In the current study, the reason for an increase in mortality in acute respiratory failure patients with vitamin D inadequacy is likely to be multifactorial. Other issues that may be important for critical illness outcomes include vitamin D related effects on vascular endothelial growth factor, endothelin and the renin-angiotensin-aldosterone system.^38–40^ Further, comorbidities including incident hypertension, glucose intolerance, the metabolic syndrome, obesity and cardiovascular disease are all associated with low 25(OH)D and higher mortality.^41–45^

The present study is not without potential limitations. Observational studies may be limited by bias, confounding, and/or reverse causation.^46^ Importantly, causality cannot be determined in our study. Our utilisation of ICD-9 and CPT coding likely reflects the measured incidence of acute respiratory failure in the cohort rather than the actual incidence. Ascertainment bias may exist in our study as the patient cohort under study had vitamin D status measurements for reasons that may be absent in other critically ill patients. Despite adjustment for multiple potential confounders, residual confounding may contribute to the observed differences in outcomes and vitamin D status could simply be a marker of baseline healthy behaviours. Specifically, we are unable to adjust for immobilisation, excessive alcohol intake, smoking status, genetic factors, hypertension, low-density lipoprotein-cholesterol, education level and low milk consumption all of which can alter 25(OH)D.^47^ ^48^ The percentage of cohort patients who are female is 55% while the percentage of African-Americans is only 6%, which may limit generalisability. The 25(OH)D-mortality association appears to be preserved when 25(OH)D is obtained within 30 days of admission. Despite this observation, vitamin D levels at the time of hospitalisation may be different from the levels when prehospital values were drawn. We also do not have any information as to why 25(OH)D concentrations were obtained in the cohort. Finally, our observations may represent a healthy user effect rather than causality.^49^

The present study has several strengths. To the best of our knowledge, our study is the first large sample study to evaluate an association between prehospital vitamin D and incident acute respiratory failure. Crucially, we have sufficient statistical power to detect a clinically relevant difference in acute respiratory failure between the vitamin D groups. The exclusion of ICD-9 codes related to heart failure and requirement of mechanical ventilation ICD-9 codes for our exposure increases the specificity of the codes for ARDS.^24^ The Deyo-Charlson Index allowed us to account for chronic medical comorbidities. Finally, by measuring vitamin D status at least 7 days prior to hospitalisation, we attempted to uncouple the influence of acute illness and inflammation on 25(OH)D levels.

## Conclusion

In summary, prehospital vitamin D inadequacy (25(OH)D <20 ng/mL) is associated with incident acute respiratory failure during critical illness and death in patients with acute respiratory failure. Despite our observations, supplementation of vitamin D in critically ill patients cannot be advocated for prevention or treatment of acute respiratory failure as our study does not establish causation. The data presented in combination with the hypothesis generating VITdAL-ICU trial data^11^ does provide an impetus to perform randomised controlled trials to determine whether vitamin D supplementation therapy might have benefit in decreasing the incidence of or improving outcomes of ARDS in critically ill patients with low 25(OH)D.

## References

[1] Rubenfeld GD, Caldwell E, Peabody E Incidence and outcomes of acute lung injury. N Engl J Med 2005;353:1685–93. doi:10.1056/NEJMoa050333 16236739 10.1056/NEJMoa050333

[2] Vincent JL, Sakr Y, Ranieri VM Epidemiology and outcome of acute respiratory failure in intensive care unit patients. Crit Care Med 2003;31 (4 Suppl):S296–9. doi:10.1097/01.CCM.0000057906.89552.8F 12682455 10.1097/01.CCM.0000057906.89552.8F

[3] Lee CM, Fan E ICU-acquired weakness: what is preventing its rehabilitation in critically ill patients? BMC Med 2012;10:115 doi:10.1186/1741-7015-10-115 23033976 10.1186/1741-7015-10-115 PMC3520774

[4] Stefan MS, Shieh MS, Pekow PS Epidemiology and outcomes of acute respiratory failure in the United States, 2001 to 2009: a national survey. J Hosp Med 2013;8:76–82. doi:10.1002/jhm.2004 23335231 10.1002/jhm.2004 PMC3565044

[5] Adams JS, Hewison M Unexpected actions of vitamin D: new perspectives on the regulation of innate and adaptive immunity. Nat Clin Pract Endocrinol Metab 2008;4:80–90. doi:10.1038/ncpendmet0716 18212810 10.1038/ncpendmet0716 PMC2678245

[6] Quraishi SA, Litonjua AA, Moromizato T Association between prehospital vitamin D status and hospital-acquired bloodstream infections. Am J Clin Nutr 2013;98:952–9. doi:10.3945/ajcn.113.058909 23945717 10.3945/ajcn.113.058909 PMC3778865

[7] Moromizato T, Litonjua AA, Braun AB Association of low serum 25-hydroxyvitamin D levels and sepsis in the critically ill. Crit Care Med 2014;42:97–107. doi:10.1097/CCM.0b013e31829eb7af 23982028 10.1097/CCM.0b013e31829eb7af

[8] Liu X, Nelson A, Wang X Vitamin D modulates prostaglandin E2 synthesis and degradation in human lung fibroblasts. Am J Respir Cell Mol Biol 2014;50:40–50. 23941558 10.1165/rcmb.2013-0211OC

[9] Dancer RCA, Parekh D, Lax S Vitamin D deficiency contributes directly to acute respiratory distress syndrome. Thorax Published Online First: 22 Apr 2015. doi:10.1136/thoraxjnl-2014-206680 10.1136/thoraxjnl-2014-206680 PMC4484044 25903964

[10] Girgis CM, Clifton-Bligh RJ, Hamrick MW The roles of vitamin D in skeletal muscle: form, function, and metabolism. Endocr Rev 2013;34:33–83. doi:10.1210/er.2012-1012 23169676 10.1210/er.2012-1012

[11] Amrein K, Schnedl C, Holl A Effect of high-dose vitamin D3 on hospital length of stay in critically ill patients with vitamin D deficiency: the VITdAL-ICU randomized clinical trial. JAMA 2014;312:1520–30. doi:10.1001/jama.2014.13204 25268295 10.1001/jama.2014.13204

[12] Ginde AA, Liu MC, Camargo CA Jr Demographic differences and trends of vitamin D insufficiency in the US population, 1988–2004. Arch Intern Med 2009;169:626–32. doi:10.1001/archinternmed.2008.604 19307527 10.1001/archinternmed.2008.604 PMC3447083

[13] Braun A, Chang D, Mahadevappa K Association of low serum 25-hydroxyvitamin D levels and mortality in the critically ill*. Crit Care Med 2011;39:671–7. doi:10.1097/CCM.0b013e318206ccdf 21242800 10.1097/CCM.0b013e318206ccdf PMC3448785

[14] Braun AB, Gibbons FK, Litonjua AA Low serum 25-hydroxyvitamin D at critical care initiation is associated with increased mortality*. Crit Care Med 2012;40:63–72. doi:10.1097/CCM.0b013e31822d74f3 21926604 10.1097/CCM.0b013e31822d74f3 PMC4427350

[15] Braun AB, Litonjua AA, Moromizato T Association of low serum 25-hydroxyvitamin D levels and acute kidney injury in the critically ill. Crit Care Med 2012;40:3170–9. doi:10.1097/CCM.0b013e318260c928 22975885 10.1097/CCM.0b013e318260c928

[16] Quraishi SA, Bittner EA, Blum L Prospective study of vitamin D status at initiation of care in critically ill surgical patients and risk of 90-day mortality. Crit Care Med 2014;42:1365–71. doi:10.1097/CCM.0000000000000210 24557421 10.1097/CCM.0000000000000210 PMC4064717

[17] Ginde AA, Camargo CA Jr., Shapiro NI Vitamin D insufficiency and sepsis severity in emergency department patients with suspected infection. Acad Emerg Med 2011;18:551–4. doi:10.1111/j.1553-2712.2011.01047.x 21518095 10.1111/j.1553-2712.2011.01047.x

[18] Venkatram S, Chilimuri S, Adrish M Vitamin D deficiency is associated with mortality in the medical intensive care unit. Crit Care 2011;15:R292 doi:10.1186/cc10585 22152332 10.1186/cc10585 PMC3388639

[19] McKinney JD, Bailey BA, Garrett LH Relationship between vitamin D status and ICU outcomes in veterans. J Am Med Dir Assoc 2011;12:208–11. doi:10.1016/j.jamda.2010.04.004 21333923 10.1016/j.jamda.2010.04.004

[20] Matthews LR, Ahmed Y, Wilson KL Worsening severity of vitamin D deficiency is associated with increased length of stay, surgical intensive care unit cost, and mortality rate in surgical intensive care unit patients. Am J Surg 2012;204:37–43. doi:10.1016/j.amjsurg.2011.07.021 22325335 10.1016/j.amjsurg.2011.07.021 PMC3992708

[21] Arnson Y, Gringauz I, Itzhaky D Vitamin D deficiency is associated with poor outcomes and increased mortality in severely ill patients. QJM 2012;105:633–9. doi:10.1093/qjmed/hcs014 22331959 10.1093/qjmed/hcs014

[22] Lee P, Eisman JA, Center JR Vitamin D deficiency in critically ill patients. N Engl J Med 2009;360:1912–14. doi:10.1056/NEJMc0809996 19403914 10.1056/NEJMc0809996

[23] Amrein K, Zajic P, Schnedl C Vitamin D status and its association with season, hospital and sepsis mortality in critical illness. Crit Care 2014;18:R47 doi:10.1186/cc13790 24661739 10.1186/cc13790 PMC4057427

[24] Cooke CR, Erickson SE, Eisner MD Trends in the incidence of noncardiogenic acute respiratory failure: the role of race. Crit Care Med 2012;40:1532–8. doi:10.1097/CCM.0b013e31824518f2 22511134 10.1097/CCM.0b013e31824518f2 PMC3329645

[25] Ross AC, Manson JE, Abrams SA The 2011 report on dietary reference intakes for calcium and vitamin D from the Institute of Medicine: what clinicians need to know. J Clin Endocrinol Metab 2011;96:53–8. doi:10.1210/jc.2010-2704 21118827 10.1210/jc.2010-2704 PMC3046611

[26] Rapoport J, Gehlbach S, Lemeshow S Resource utilization among intensive care patients. Managed care vs traditional insurance. Arch Intern Med 1992;152:2207–12. doi:10.1001/archinte.1992.00400230033006 1444680

[27] Charlson ME, Pompei P, Ales KL A new method of classifying prognostic comorbidity in longitudinal studies: development and validation. J Chronic Dis 1987;40:373–83. doi:10.1016/0021-9681(87)90171-8 3558716 10.1016/0021-9681(87)90171-8

[28] Martin GS, Mannino DM, Eaton S The epidemiology of sepsis in the United States from 1979 through 2000. N Engl J Med 2003;348:1546–54. doi:10.1056/NEJMoa022139 12700374 10.1056/NEJMoa022139

[29] Duncan DT, Sharifi M, Melly SJ Characteristics of walkable built environments and BMI z-scores in children: evidence from a large electronic health record database. Environ Health Perspect 2014;122:1359–65. 25248212 10.1289/ehp.1307704 PMC4256697

[30] Singh GK Area deprivation and widening inequalities in US mortality, 1969–1998. Am J Public Health 2003;93:1137–43. doi:10.2105/AJPH.93.7.1137 12835199 10.2105/ajph.93.7.1137 PMC1447923

[31] Kind AJ, Jencks S, Brock J Neighborhood socioeconomic disadvantage and 30-day rehospitalization: a retrospective cohort study. Ann Intern Med 2014;161:765–74. doi:10.7326/M13-2946 25437404 10.7326/M13-2946 PMC4251560

[32] Poulose JT, Cartin-Ceba R, Shoja A, *et al*. Comparison of International Classification of Disease-Ninth Revision (ICD-9) coding with retrospective case review for the diagnosis of acute respiratory distress syndrome. Am J Respir Crit Care Med. 2009;179:A4660.

[33] Force ADT, Ranieri VM, Rubenfeld GD Acute respiratory distress syndrome: the Berlin Definition. JAMA 2012;307: 2526–33. 22797452 10.1001/jama.2012.5669

[34] Parsons PE, Eisner MD, Thompson BT Lower tidal volume ventilation and plasma cytokine markers of inflammation in patients with acute lung injury. Crit Care Med 2005;33:1–6; discussion 230–2 doi:10.1097/01.CCM.0000149854.61192.DC 15644641 10.1097/01.ccm.0000149854.61192.dc

[35] Piantadosi CA, Schwartz DA The acute respiratory distress syndrome. Ann Intern Med 2004;141:460–70. doi:10.7326/0003-4819-141-6-200409210-00012 15381520 10.7326/0003-4819-141-6-200409210-00012

[36] Wang TT, Nestel FP, Bourdeau V Cutting edge: 1,25-dihydroxyvitamin D3 is a direct inducer of antimicrobial peptide gene expression. J Immunol 2004;173:2909–12. doi:10.4049/jimmunol.173.5.2909 15322146 10.4049/jimmunol.173.5.2909

[37] Zanetti M Cathelicidins, multifunctional peptides of the innate immunity. J Leukoc Biol 2004;75:39–48. doi:10.1189/jlb.0403147 12960280 10.1189/jlb.0403147

[38] Cardus A, Parisi E, Gallego C 1,25-Dihydroxyvitamin D3 stimulates vascular smooth muscle cell proliferation through a VEGF-mediated pathway. Kidney Int 2006;69:1377–84. 16557229 10.1038/sj.ki.5000304

[39] Wu J, Garami M, Cheng T 1,25(OH)2 vitamin D3, and retinoic acid antagonize endothelin-stimulated hypertrophy of neonatal rat cardiac myocytes. J Clin Invest 1996;97:1577–88. doi:10.1172/JCI118582 8601621 10.1172/JCI118582 PMC507220

[40] Forman JP, Williams JS, Fisher ND Plasma 25-hydroxyvitamin D and regulation of the renin-angiotensin system in humans. Hypertension 2010;55:1283–8. doi:10.1161/HYPERTENSIONAHA.109.148619 20351344 10.1161/HYPERTENSIONAHA.109.148619 PMC3023301

[41] Forman JP, Giovannucci E, Holmes MD Plasma 25-hydroxyvitamin D levels and risk of incident hypertension. Hypertension 2007;49:1063–9. doi:10.1161/HYPERTENSIONAHA.107.087288 17372031 10.1161/HYPERTENSIONAHA.107.087288

[42] Chonchol M, Scragg R 25-Hydroxyvitamin D, insulin resistance, and kidney function in the Third National Health and Nutrition Examination Survey. Kidney Int 2007;71:134–9. doi:10.1038/sj.ki.5002002 17082756 10.1038/sj.ki.5002002

[43] Ford ES, Ajani UA, McGuire LC Concentrations of serum vitamin D and the metabolic syndrome among U.S. adults. *Diabetes care*. 2005;28:1228–30. 10.2337/diacare.28.5.1228 15855599

[44] Wortsman J, Matsuoka LY, Chen TC Decreased bioavailability of vitamin D in obesity. Am J Clin Nutr 2000;72:690–3. 10966885 10.1093/ajcn/72.3.690

[45] Anderson JL, May HT, Horne BD Relation of vitamin D deficiency to cardiovascular risk factors, disease status, and incident events in a general healthcare population. Am J Cardiol 2010;106:963–8. doi:10.1016/j.amjcard.2010.05.027 20854958 10.1016/j.amjcard.2010.05.027

[46] Ho PM, Peterson PN, Masoudi FA Evaluating the evidence: is there a rigid hierarchy? Circulation 2008;118:1675–84. doi:10.1161/CIRCULATIONAHA.107.721357 18852378 10.1161/CIRCULATIONAHA.107.721357

[47] Wang TJ, Zhang F, Richards JB Common genetic determinants of vitamin D insufficiency: a genome-wide association study. Lancet 2010;376:180–8. doi:10.1016/S0140-6736(10)60588-0 20541252 10.1016/S0140-6736(10)60588-0 PMC3086761

[48] Forrest KY, Stuhldreher WL Prevalence and correlates of vitamin D deficiency in US adults. Nutr Res 2011;31:48–54. doi:10.1016/j.nutres.2010.12.001 21310306 10.1016/j.nutres.2010.12.001

[49] Shrank WH, Patrick AR, Brookhart MA Healthy user and related biases in observational studies of preventive interventions: a primer for physicians. J Gen Intern Med 2011;26:546–50. doi:10.1007/s11606-010-1609-1 21203857 10.1007/s11606-010-1609-1 PMC3077477

